# Can a general factor be derived from employees’ responses to items on the Individual Work Performance Review?

**DOI:** 10.4102/ajopa.v6i0.133

**Published:** 2024-01-22

**Authors:** Xander van Lill, Leoni van der Vaart

**Affiliations:** 1 Department of Industrial Psychology and People Management, College of Business and Economics, University of Johannesburg, Johannesburg, South Africa; 2 Department of Product and Research, JVR Africa Group, Johannesburg, South Africa; 3 Consulting, Peter Berry Consultancy, Sydney, Australia; 4 Opentia Research Unit, School of Industrial Psychology and Human Resource Management, Economic and Management Sciences, North-West University, Vanderbijlpark, South Africa; 5 Department of Psychology, Norwegian University of Science and Technology, Trondheim, Norway

**Keywords:** general performance factor, generic performance, individual work performance, exploratory structural equation modelling, bifactor model, Individual Work Performance Review

## Abstract

This study aimed to investigate whether permissible inferences can be derived from employees’ standing on a general performance factor from their responses to the Individual Work Performance Review (IWPR) items. The performance of 448 employees was rated (by their managers) using the IWPR. Latent variable modelling was performed through a bifactor exploratory structural equation model with the robust version of the maximum likelihood estimator. The general factor’s score was also used to inspect correlations with two work performance correlates: tenure and job level. In line with international findings, the results suggested that a general factor could explain 65% of the common variance in the 80 items of the IWPR. Job level, but not tenure, correlated with general job performance. The results support calculating an overall score for performance, which might be a suitable criterion to differentiate top performers, conduct criterion validity studies, and calculate the return on investment of selection procedures or training programmes.

**Contribution:**

The present study provides initial evidence for a general factor influencing employees’ responses to items on a generic performance measure in South Africa. In addition, the study showcases the application of advanced statistical methods in factor analyses, demonstrating their efficacy in evaluating the psychometric properties of hierarchical factor models derived from data provided on performance measures.

## Introduction

Rigorous measurement of individual work performance is critical for the effective functioning of organisations. Organisations stand to benefit when desirable work behaviours or objective work outcomes, are clarified and reinforced based on reliable, valid, and unbiased performance data (Aguinis, 2019). In the context of an organisation, desirable behaviours include acts that assist the system in achieving its collective goals, and ultimately ensure its survival in a competitive business landscape (Campbell & Wiernik, 2015). Business survival is, of course, also dependent on the strategic relevance of the organisation’s products and/or services to customers (Drucker & Maciariello, 2008).

More so than other workplace outcomes, individual work performance can be seen as a fundamental building block of the effectiveness with which organisations execute strategy, which, in turn, generates revenue (Campbell & Wiernik, 2015). As stated by Campbell and Wiernik (2015):

[*O*]ther dependent variables are extremely important, including individual work satisfaction, commitment, engagement, stress/health, and work/family balance. However, without individual performance, there can be no job to be satisfied with, no organization to be committed to, and no work to balance with family. (p. 48)

Therefore, scholars and practitioners must thoroughly understand performance (Campbell & Wiernik, 2015).

Performance can be conceptualised as a multidimensional construct that comprises broad domains such as in-role-, extra-role-, adaptive-, leadership-, and counterproductive performance. The broad domains can be broken down into narrower dimensions. For example, in-role performance can be broken down into quality of work, quantity of work, rule adherence, and technical performance. A multidimensional view of performance is valuable when tailoring feedback for individual development (Campbell & Wiernik, 2015). However, in the development and validation of actuarial selection procedures, an overall score might be a meaningful way to determine the relative importance of different determinants of work performance (Aguinis, 2019). McNeish and Wolf (2020) also acknowledge that, in practice, it is common to calculate overall sum scores to guide decisions about people based on psychometric constructs. Some scholars (e.g. Rodriguez et al., 2016) argue that unit-weighted total scores (or summed total scores) may be justified in situations where a general factor explains a significant amount of the common variance in the items of interest, independent of the group factors.

Currently, there is a paucity of empirical literature supporting a general factor of performance among generic measures of individual work performance in South Africa (Van Lill & Taylor, 2022). Consequently, human resource (HR) professionals would be hard-pressed to justify using behaviour-based overall performance scores in predictive studies and making high-stakes decisions. This is not to say that an outcome-based measure of performance, especially an overall economy-based performance score such as the number of sales, requires evidence of a general (or global) factor. However, evidence supporting a general factor is important in performance measures focusing on observed behaviour (Viswesvaran et al., 2005).

Viswesvaran et al. (2005) argue that identifying a substantial general factor influencing job performance has noteworthy ramifications for the methodology of criterion measurement in validation studies. More specifically, it suggests that the traditional approach of consolidating component measures of job performance to construct an overarching metric of overall job performance, as commonly practised in many primary validity investigations and validity generalisation studies, is theoretically and empirically sound. Therefore, this study aimed to formally test whether the data are best explained by a general factor underlying all the items in the Individual Work Performance Review (IWPR) (Van Lill & Taylor, 2022) in addition to the specific performance factors. In other words, the aim was to test whether a (‘quantitative’) global factor exists – in addition to the ‘qualitatively’ different narrow dimensions – that reflects how well an individual is performing. It was envisioned that empirical evidence from such an investigation could support the calculation of a total score based on the narrow dimensions of performance while using the IWPR (Campbell & Wiernik, 2015).

A general factor is not proposed to replace the 5 broad or even the 20 narrow dimensions in the IWPR. Instead, the aim was to provide an additional layer of interpretation of individual work performance, especially when high-stake decisions need to be made. This study also aimed to confirm the criterion validity of the final factor-analytic solution by using biographical variables (i.e. tenure and job level) relevant to a general factor of performance. This study, therefore, aims to contribute to the evidence surrounding the structural and criterion validity of the IWPR in South Africa.

### Hierarchical structure of a general factor of individual work performance

Sound explanations of employees work performance provide HR and industrial psychologists with the opportunity to enhance their performance through two main types of interventions, namely flow and stock interventions. Selection, a crucial element of flow interventions, has been a research focus since Paul Meehl’s pioneering work in the 1960s (Meehl, 1954). Subsequent studies comparing clinical and statistical selection procedures have consistently favoured mechanical actuarial methods, indicating a preference for these in practice. When constructing an actuarial prediction model, the typical approach involves favouring the regression of a single criterion measure against a weighted composite of predictors (Cascio & Aguinis, 2019). It’s worth observing that while it’s feasible to develop and validate actuarial selection procedures using multiple criteria, there isn’t a widely recognised procedure for assessing the fairness and utility of selection methods employing multiple criteria.

Creating a single criterion measure can be achieved using a composite criterion, which involves adding up a weighted or unweighted combination of performance dimension scores. However, proponents of the multiple criterion approach rightfully critique this method, emphasising that the distinct first-order individual work performance factors cannot be logically combined. To illustrate this point, Cattell (1957, p. 11) aptly stated, ‘Ten men and two bottles of beer cannot be added to give the same total as two men and ten bottles of beer’. When employing multiple criteria to assess work performance, each employee is represented as a point in a multidimensional criterion space, and work success is defined within a smaller subspace of that multidimensional framework. In contrast, advocates of the composite criterion approach argue that even when using multiple criteria, making selection decisions for applicants falling within the success subspace still requires combining the separate criterion estimates into a single score (Cascio & Aguinis, 2019).

Another way to get to a single criterion score is to measure overall work performance without calculating it from the dimension scores. Individual work performance could be viewed as a hierarchical model, with a general factor that, in turn, breaks down into narrower performance dimensions (Viswesvaran et al., 2005). Both higher-order and bifactor models represent hierarchical factor models (Morin, 2023). In the context of this study, the narrow factors, such as quality of work, mediate in connecting the observed variables (or items) to the general performance factor in the higher-order model. However, it is important to observe that general performance factor in this model does not account for unique variance in the observed variables beyond what the narrow factors explain. Bifactor models offer a contrasting perspective. In these models, the orthogonal broad performance factor explains unique variance in the observed variables independently of the variance accounted for by the orthogonal narrow factors (Gignac, 2016), the preferred hierarchical model in the present study.

The IWPR was purposefully based on prior generic models of performance, such as that of Koopmans et al. (2013) and Viswesvaran et al. (2005), which either theoretically alluded to or empirically demonstrated the existence of a general factor of performance. One possible explanation for a general factor is that employees generally have a good idea of what’s expected of them to be considered good at their jobs. However, how well they meet these expectations can vary depending on certain factors. Some employees who score high on cognitive ability and personality-based integrity tend to perform well in all aspects of their job. By contrast, employees who score lower on these factors tend to perform less well in all areas of their job. This difference in how well employees do their jobs causes the different aspects of job performance to be connected.

The general factor reflects pro-organisational behaviour that aids the achievement of collective goals. Narrower dimensions, by contrast, reflect more specific ways in which employees contribute to organisational effectiveness. Compared with a general factor, narrower dimensions are qualitatively more meaningful during performance feedback. Performance feedback on narrower dimensions is more likely to allow the derivation of actionable steps that employees could take to increase their overall performance at work. General performance, or giving an employee just an overall quantitative score, might be perceived as too ambiguous and less meaningful from a performance development perspective (Carpini et al., 2017). However, many narrower dimensions of performance might be jarring in larger decision-making processes when, for example, managers must make administrative decisions about rewarding and promoting employees (stock interventions). Overall performance scores are, therefore, easier to employ for administrative decisions (Aguinis, 2019). The researchers of this study do not aim to refute the relevance of such decisions on multiple criteria. Still, they want to investigate whether using overall performance scores is merited.

The need to differentiate employees based on an overall performance score is especially salient given the distribution of overall performance in organisations. A small number of star employees contribute disproportionately to an organisation’s overall effectiveness, making it essential to identify and retain such individuals. Differentiating high performers makes it critical for organisations to use an encompassing, valid, reliable, and unbiased quantitative score in decision-making processes (Aguinis & O’Boyle, 2014). An overall performance score could be a more manageable variable when considering its impact on more distal, unit-level outcomes. This could include calculating return on investment (ROI), given increases in performance for a group of individuals (Schleicher et al., 2019; Seland & Theron, 2021). Overall performance scores are further considered important criterion variables studied in the workplace and are often used to determine the utility of selection and development initiatives (Aguinis, 2019; Campbell & Wiernik, 2015; Viswesvaran et al., 2005). At a unit level, the utility of these procedures often requires that predictive studies are conducted to determine the impact (statistical effects) of selection procedures or training initiatives on future job performance. For example, when determining the utility of selection procedures and training programmes, two overall performance-related metrics are required to calculate return on investment, namely (Cascio & Boudreau, 2011):

Sdy the standard deviation of overall performance in monetary value, and

r the effect size of a selection procedure or the difference between overall performance before and after training.

Overall performance scores are, therefore, instrumental to larger strategic decisions that must be made about people practices within organisations. Monitoring these trends in organisations justifies the continued investment and adjustment of selection and development procedures aimed at ensuring a competitive staff complement (Cascio & Boudreau, 2011).

The investigation of a general factor of individual work performance hinges, in part, on a careful selection of generic performance dimensions. Generic performance dimensions reflect actions independent of specific jobs (Harari & Viswesvaran, 2018) that facilitate achieving organisational goals (Campbell & Wiernik, 2015). A common problem associated with job-specific performance measures is the clinical or intuitive creation and assignment of performance criteria across jobs, making it harder to aggregate scores across employees in organisations. This inevitably erodes the sample sizes (statistical power) required to investigate a general factor (Myburgh, 2013). Notable work in the domain of generic performance models in South Africa to date includes Schepers’ (2008) development of the Work Performance Questionnaire (WPQ), Myburgh’s (2013) Generic Performance Questionnaire (GPQ), Van Der Vaart’s (2021) validation of the internationally developed Individual Work Performance Questionnaire (IWPQ) (Koopmans et al., 2013), and Van Lill and Taylor’s (2022) development of the IWPR.^1^

While all the generic work performance measures validated for South Africa showed that broad and/or narrow performance dimensions covary, no researchers, except Van Der Vaart (2021), empirically tested a hierarchical model with a single, general performance factor. His findings did not support the hierarchical model, but the decision was based only on fit indices. Research indicates that the decision to retain such a model for subsequent analysis should not be based solely on fit indices but should also consider alternative model parameters (i.e. cross-loadings and inter-factor correlations) (Morin et al., 2016, 2020).

Viswesvaran (1993) was the first to notice and argue for a general factor of individual work performance. Two arguments are forwarded in support of the general factor. Firstly, meaningful predictors of general performance, such as cognitive ability and personality-based integrity, appear more hierarchical. A general factor of mental ability seems to explain the variance between specific cognitive aptitudes (Schneider & McGrew, 2018). Personality-based integrity is a composite trait, like meta-trait stability, which explains the variance between conscientiousness, agreeableness, and emotional stability (DeYoung, 2015). Given the proportional variance that cognitive ability (*p* = 0.31) and personality-based integrity (*p* = 0.31) explain in overall performance scores (Sackett et al., 2022), it is plausible that a general factor might also exist in performance. Secondly, it appears that contextual performance (extra-role performance or organisational citizenship behaviours) positively affects the rating of other performance dimensions in the same direction (Viswesvaran et al., 2005). Individuals who are highly motivated to go beyond what is required, reflected by directed, high-intensity, and persistent work effort, might also receive higher scores on other performance dimensions.

Critique expressed against a general factor among performance dimensions suggests that the general factor could be attributed to a statistical artefact brought about by the halo effect (Holzbach, 1978; Landy et al., 1982). Halo errors reflect the overall positive impression of an employee’s performance on one or more dimensions, and tend to negatively skew results on other scales towards also being more positive (Aguinis, 2019). However, a meta-analytical study conducted by Viswesvaran et al. (2005) revealed that a general factor, after controlling for halo error and three other sources of measurement error, explained 60% of the total variance at the construct level. Harari and Viswesvaran (2018) argue that it is, therefore, appropriate to conceptualise individual work performance as a hierarchical model, with a general performance factor at the model’s apex.

The replicability of a general factor of performance in South Africa was tested by employing the narrow dimensions of the IWPR. Definitions of the narrow dimensions are provided in Table 1. The definitions were derived from a literature review conducted on generic dimensions of individual performance and obtained with permission from Van Lill and Taylor (2022). Their study supports the structural validity of the narrow dimensions. The narrow dimensions displayed covariation, which suggests the presence of a general factor.

**TABLE 1:** Definitions of the narrow performance factors on the Individual Work Performance Review.

Dimension	Definition
Quality of work	The thoroughness with which employees perform work tasks, evident in the degree to which employees pay attention to detail and minimise errors
Quantity of work	How productive employees are in meeting challenging work goals in terms of both the volume of output and meeting the required time frame
Rule adherence	Employees’ tendency to comply with informal and formal rules and regulations of the organisation
Technical performance	The degree to which employees perform well at tasks that are differentiated, complicated, and require a certain level of expertise
Helpful behaviours	Employees’ acts of kindness towards co-workers
Taking initiative	Demonstrated by employees showing self-starting behaviour and doing more than is expected of them.
Self-development	Reflected in employees’ initiatives to enhance their competence by actively gaining knowledge and learning new skills that could benefit the team
Innovative behaviours	Employees exploring or generating new opportunities and implementing new and creative ideas
Emotional resilience	Demonstrated when employees maintain their composure when they have to work under high pressure
Dealing with complexity	Demonstrated when employees think, decide, and act sensibly under uncertain and unusual situations when there are no clear guidelines
Adapting to crises	The degree to which employees remain objective, make swift decisions, and react with appropriate urgency to a crisis
Interpersonal flexibility	Reflected in how comfortable employees are with situations in which people with diverse views do not agree with each other; it is also represented by employees’ open-mindedness in interaction with co-workers from different backgrounds
Task-orientated leadership	Demonstrated by employees when they direct the efforts of co-workers towards the achievement of team goals
Relations-orientated leadership	Demonstrated when consideration is used to empower and motivate co-workers to achieve team goals
Change-orientated leadership	The degree to which employees inspire their co-workers to effect required changes to the way they do their work
Network-orientated leadership	The degree to which networking is used to connect co-workers with key role players inside and outside the organisation
Interpersonal rudeness	Disrespectful acts that reflect a lack of regard for others
Withholding effort	Demonstrated when employees show a lack of enthusiasm in their work by exerting less effort than is expected for the position they hold
Stagnation	Demonstrated when an employee displays an unwillingness to learn new skills, thereby affecting team effectiveness
Stubborn resistance	Reflected in an employee’s unreasonable opposition to change or an unwillingness to support initiatives at work, and suggests a destructive form of opposition to team goals

*Source*: Van Lill, X., & Van Der Merwe, G. (2022). Differences in self- and managerial-ratings on generic performance dimensions. *SA Journal of Industrial Psychology, 48*, 1–10. https://doi.org/10.4102/sajip.v48i0.2045

### Research objectives and hypotheses

Based on previous meta-analytical evidence in support of a general factor of performance, it was hypothesised that:

**H_1_**: A general performance dimension explains variance in the 80 items of the IWPR, independent of the variance that the narrow dimensions explain in the same set of items.

This study also sought to source evidence for the validity of the inferences derived from employees’ standing on the general performance factor. To achieve this, additional biographical variables were used, namely job level for the entire sample and tenure for a subset of the data collected for this project. Tenure, which, in this case, also reflects job experience, is related to performance independent of the complexity levels of jobs (Schmidt et al., 2016). Job level could be viewed as a proxy for job complexity, where job complexity increases as greater educational attainment is required for professional or managerial roles. More complex jobs might afford employees greater autonomy or attract job applications with higher cognitive ability, experience, and job knowledge, consequently increasing job performance (Hunter et al., 1990). In this study, the complexity of jobs was argued to increase from low to high based on the following order:

1. semi-skilled (perform skilled work that does not require advanced training)
2. skilled (perform skilled work that requires advanced training)
3. professional (perform work that requires being registered with a professional board) and management (set and drive organisational goals).

The above levels were informed by classifications of jobs into occupational categories, as reported by Statistics South Africa (2012) and the National Center for O*NET Development (2022). Job level is also likely to be positively related to job performance if valid decisions were made to select or promote employees (Hunter et al., 1990; Schmidt et al., 2016). Based on existing evidence, it was hypothesised that:

**H_2A_**: Tenure is positively related to general work performance.

**H_2B_**: Job level is positively related to general work performance.

## Method

### Study design

A cross-sectional, quantitative research design was utilised in this study. A cross-sectional design enabled a nuanced view of the multifaceted nature of self and manager ratings of performance at a single point in time, as well as an efficient quantitative exploration of relationships between a large set of variables across different organisational contexts (Spector, 2019; Van Lill & Taylor, 2022; Van Lill & Van Der Merwe, 2022).

### Participants

The researchers attempted to draw a sample from organisations in different economic sectors to increase the results’ external validity (generalisability) (Aguinis & Edwards, 2014). Fifteen organisations across several economic sectors in South Africa were invited to participate in the study. A census- or stratified sampling strategy was used to identify 448 employees from 6 organisations representing the industrial, agriculture, finance, professional services, and information technology sectors. The managers of the 448 employees were then invited to rate the performance of the representative employees via an email link. A calculation of statistical power, using computer software developed by Preacher and Coffman (2006), returned a value of unity that suggested that an incorrect model with 1690 degrees of freedom would be correctly rejected (α = 0.05; null RMSEA [root mean square error of approximation] = 0.05; alternative RMSEA = 0.08) (Van Lill & Taylor, 2022).

The employees, who the managers rated, had a mean age of 38.77 years (standard deviation = 7.02 years). Most employees self-identified as white (*n* = 201; 48%), followed by black African (*n* = 136; 30%), Indian (81; 18%), mixed race (mixed ancestry; *n* = 27; 6%), and Asian (3; 1%). More women (*n* = 249; 56%) than men (*n* = 199; 44%) participated in the study. Most of the employees were registered professionals (*n* = 142; 32%), followed by mid-level managers (*n* = 106; 24%), skilled employees (103; 23%), low-level managers (*n* = 84; 19%), semi-skilled employees (9; 2%), and top-level managers (4; 1%). The mean tenure of employees in the subset of data comprising 332 employees was 7.81 years (standard deviation = 5.67 years) (Van Lill & Taylor, 2022).

### Instruments

The IWPR was administrated to collect the data. The IWPR consists of 80 items covering 20 narrow performance dimensions. Each item was measured using a five-point behaviour frequency scale (Aguinis, 2019). Word anchors defined the extreme points of each scale, namely (1) *Never demonstrated* and (5) *Always demonstrated* (Van Lill & Van Der Merwe, 2022). The guidelines of Casper et al. (2020) were used to guide the qualitative interpretation of numeric values between the extreme points, to better approximate an interval rating scale, namely (2) *Rather infrequently demonstrated*, (3) *Demonstrated some of the time*, and (4) *Quite often demonstrated*. Narrow dimensions of the IWPR displayed good internal consistency reliability in previous research (α and ω ≥ 0.83; Van Lill & Taylor, 2022; Van Lill & Van Der Merwe, 2022).

### Procedure

Data on performance were collected by asking managers of the 448 employees to rate their employees’ performance. A study by Van Lill and Van Der Merwe (2022) revealed that employees significantly inflate self-ratings on the IWPR (Van Lill & Taylor, 2022) compared to managerial ratings, because of leniency bias. Managers might provide a more conservative and accurate estimate of work performance (Van Lill & Van Der Merwe, 2022).

At the outset of the review, the direct managers and respondents received information on the developmental purpose of the study, the nature of the measurement, voluntary participation, benefits of participation, anonymity of the data, and that their data would be used for research purposes. The University of Johannesburg granted ethical clearance for the study (reference no. IPPM-2020-455) (Van Lill & Taylor, 2022).

### Data analysis

Mplus 8.6 (Muthén & Muthén, 2021) was used to conduct the statistical analyses. Competing measurement models were tested sequentially to identify the best-fitting measurement models. The measurement models indicate the construct-relevant multidimensionality of the IWPR. Both the independent cluster model (ICM) approach to confirmatory factor analysis (CFA) and the exploratory structural equation modelling (ESEM) frameworks were used. Independent cluster model-confirmatory factor analysis is often critiqued for its restrictive assumptions (e.g. items are not allowed to load onto non-target factors), which are not feasible when modelling theoretically related constructs (Morin et al., 2020), such as performance. Exploratory structural equation modelling relaxes these assumptions and allows items to cross-load onto non-target factors (albeit these loadings are constrained to a minimum). Allowing these cross-loadings minimises the impact of biased parameter estimates (e.g. over-inflated correlations) (Howard et al., 2018).

The ESEM code generator tool of Mplus was used to generate the syntaxes for the ESEM models (De Beer & Van Zyl, 2019). Associations between the different performance facets create the possibility of an overarching factor that further explains the dimensionality of the IWPR. For this reason, hierarchical and bifactor models were specified in addition to the first-order models.

All models were estimated with the robust version of the maximum likelihood (MLR) estimator, as it is more suitable for data that are not normally distributed (Wang & Wang, 2020). The following goodness-of-fit indices (GFI) were considered for the assessment of model fit to the data: the comparative fit index (CFI), the Tucker-Lewis index (TLI), the RMSEA, and the standardised root mean square residual (SRMR). Based on standard guidelines, values greater than 0.90 for the CFI and TLI were indicators of adequate fit, whereas values smaller than 0.08 for the RMSEA and SRMR were indicators of acceptable fit (Wang & Wang, 2020).

In addition to the fit indices, which are influenced by model complexity, factor loadings, cross-loadings, and factor correlations were also considered during model evaluation (Morin et al., 2016, 2020). Discriminant validity was evaluated using the 0.80 cut-off value for the upper limit of the 95% confidence interval (95% CI) of the correlations between the different facets (Rönkkö & Cho, 2022). Following this approach, it is important to clarify what discriminant validity means in the context of this study:

Two measures intended to measure distinct constructs have discriminant validity if the absolute value of the correlation between the measures after correcting for measurement error is low enough for the measures to be regarded as measuring distinct constructs. (p. 11)

Bifactor indices, that is explained common variance (ECV), omega (ω), and omega hierarchical (ω_HS_), were also calculated using Dueber’s (2021) *R* package BifactorIndicesCalculator. These indices shed further light on the uni- versus multi-dimensionality of constructs. After identifying the best-fitting measurement model, factor scores were exported for correlational analyses in jamovi Version 2.3 (The Jamovi Project, 2022). The following cut-off criteria were used to interpret the effect sizes of the correlations: *r* = ≥ 0.10 (small effect), *r* = ≥ 0.30 (medium effect), and *r* ≥ 0.50 (large effect) (Cohen, 1992).

### Ethical considerations

At the initiation of the performance review process, comprehensive information explaining the developmental objectives of the study, the characteristics of the measurement employed, the voluntary nature of participation, the potential benefits associated with involvement, and the safeguarding of data anonymity was shared with both direct managers and participants. Explicit notification was provided, affirming that the collected data would be utilised exclusively for research purposes. Ethical clearance for the study, denoted by reference number IPPM-2020-455 and dated October 6, 2020, was duly granted by the Research Ethics Committee of the Department of Industrial Psychology and People Management at the University of Johannesburg.

## Results

Table 2 contains the GFI for each of the measurement models. Models 1 to 3 were the ICM-CFA versions of the measurement model. In Model 1, all items were allowed to load onto their a priori determined factors (see Table 1). The 20 performance factors (or facets) were allowed to correlate. This model is also termed the ‘correlated traits’ model (Reise et al., 2010). In Model 2, a second-order hierarchical CFA model was specified, in which the items were loaded onto the 20 factors and a higher-order (performance) factor. In this model, a measurement structure is placed onto the correlations between the factors, translating into the ‘higher-order’ dimension, explaining why the ‘lower-order’ dimensions are related (Reise et al., 2010). Here, the loading of each item on the first-order factor is multiplied by the loading of the lower-order factor on the higher-order factor to represent the indirect effect of the higher-order factor on the item. The loading of the lower-order factor onto the higher-order factor is a constant (and thus constrained) for all indicators associated with a specific lower-order factor (Morin et al., 2020). Similarly, the variance in the item unique to the lower-order factor is also constrained for all items associated with a specific lower-order factor (Morin, 2023).

**TABLE 2:** Model fit statistics for the competing measurement models (*n* = 448).

Model	χ^2^	*df*	RMSEA	90% CI	CFI	TLI	SRMR	AIC	BIC
ICM-CFA	5395.74*	2890	0.044	0.042, 0.046	0.93	0.92	0.05	59672.86	61437.93
H-CFA	6771.23*	3050	0.052	0.051, 0.054	0.89	0.89	0.08	60970.42	62078.71
Bifactor-CFA	6872.73*	3000	0.054	0.052, 0.055	0.89	0.88	0.07	61173.62	62487.15
ESEM	3243.60*	1750	0.044	0.041, 0.046	0.96	0.92	0.01	58913.06	65357.58
H-ESEM	3143.43*	1940	0.037	0.035, 0.040	0.97	0.95	0.01	58564.18	64228.79
Bifactor-ESEM	3130.33*	1690	0.044	0.041, 0.046	0.96	0.92	0.01	58836.71	65527.52

ESEM, exploratory structural equation modelling; H-ESEM, hierarchical ESEM; ICM-CFA, independent cluster model-confirmatory factor analysis; *df*, degrees of freedom; χ^2^, Chi-square; CFI, comparative fit index; TLI, Tucker-Lewis index; RMSEA, root mean square error of approximation; CI, confidence interval; SRMR, standardised root mean square residual; AIC, Akaike information criterion; BIC, Bayes information criterion.

* , *p* < 0.001.

Model 3 was similar to Model 2,^2^ except that the items were allowed to load directly onto a general (performance) factor instead of being mediated by their own primary factors, resulting in an empirical bifactor-CFA model. In addition to loading onto the general factor, the items were allowed to load only onto one (i.e. their own) primary factor, resulting in a ‘restricted’ (or confirmatory) bifactor model (Reise et al., 2010). Model 3 is considered a hierarchical model (like Model 2), as the general factor is the first-order factor (Gignac, 2016). In both these models, neither the general (or higher-order) and specific (or lower-order) factors, nor the specific (or lower-order) factors were allowed to correlate. This allows one to quantify the proportion of variance that is shared across all items (and captured by the general or higher-order factor) and the variance that is unique to each item (and captured by the specific or lower-order factors) (Morin et al., 2020). The remaining models (Models 4 to 6) were specified using ESEM principles. Target rotation (relying on the a priori-specification of the key construct indicators such as with CFA approaches) was used for Model 4, whereas orthogonal rotation was used for Models 5 and 6. Models 4 to 6 differ from Models 1 to 3 only in that items were allowed to cross-load, but these cross-loadings were targeted to be as close to zero as possible (Morin et al., 2020).

Model selection commenced with a comparison between the ICM-CFA (i.e. Model 1) and ESEM (i.e. Model 4) solutions, as recommended by Morin (2023). Although the 20-factor CFA and the ESEM solutions fit the data well, the ESEM solution performed slightly better (i.e. higher CFI value and lower SRMR value). Table S1 (Supplementary file can be obtained at https://osf.io/azvkb/?view_only=21cc74cc5ebd443fa6a9dac183ce0116) provides the factor loadings for the ICM-CFA and ESEM solutions. As expected, the average factor loadings in the ICM-CFA (|λ| = 0.55 to 0.95; *M* = 0.87) solution were higher than those in the ESEM (|λ| = 0.22 to 0.90; *M* = 0.65) solution.

Regardless of the drop in factor loadings, the specific factors in the ESEM solution were well-defined and corresponded to the theoretically proposed relations between the items and the facets. In the ESEM solution, the target facet loadings were higher than the cross-loadings, which were generally very small^3^ (|λ| = -0.27 to 0.32; *M* = 0.01). Significant cross-loadings further supported the choice of the ESEM instead of the ICM-CFA model (cf. Morin et al., 2016, 2020). The factor correlations reported in Table 3 were smaller in the ESEM solution than in the ICM-CFA solution. They were also all in the expected direction, and most were significant. These various considerations (i.e. model fit, well-defined facets, and significant cross-loadings) led to the retention of the ESEM solution. The upper limit of the 95% CIs for the factor correlations ranged from –0.44 to 0.79, suggesting that all subscales displayed sufficient discriminant validity (Rönkkö & Cho, 2022).

**TABLE 3:** Latent factor correlations from the 20-factor confirmatory factor analysis (under the diagonal) and exploratory structural equation modelling (above the diagonal) solutions.

Dimension	QLW	QNW	REA	TNP	HPB	TII	SFD	IOB	ETR	DLC	APC	IEF	IER	WHE	SGN	SBR	TKL	RAL	CNL	NWL
QLW	-	0.48^a^	0.40^c^	0.48^c^	0.23^c^	0.36^c^	0.26^c^	0.15^a^	0.34^c^	0.39^c^	0.37^c^	0.24^c^	−0.16^b^	−0.40^c^	−0.22^c^	−0.18^a^	0.22^c^	0.20^b^	0.17^a^	0.19^b^
QNW	0.87^c^	-	0.44^c^	0.35^a^	0.30^a^	0.45^b^	0.23	0.12	0.22	0.28	0.29^a^	0.18	−0.10	−0.40	−0.24	−0.07	0.27	0.20^a^	0.17	0.22
REA	0.71^c^	0.76^c^	-	0.35^c^	0.46^c^	0.41^c^	0.27^c^	0.12	0.37^c^	0.31^c^	0.37^c^	0.49^c^	−0.38^c^	−0.37^b^	−0.32^c^	−0.29^c^	0.31^c^	0.42^c^	0.30^c^	0.27^c^
TNP	0.69^c^	0.67^c^	0.53^c^	-	0.34^c^	0.49^c^	0.54^c^	0.46^c^	0.40^c^	0.63^c^	0.55^c^	0.33^c^	−0.18^b^	−0.25^a^	−0.35^c^	−0.16	0.37^c^	0.26^c^	0.49^c^	0.52^c^
HPB	0.54^c^	0.63^c^	0.69^c^	0.52^c^	-	0.48^c^	0.42^c^	0.30^c^	0.32^c^	0.34^c^	0.38^c^	0.53^c^	−0.37^c^	−0.32^b^	−0.32^c^	−0.28^c^	0.38^c^	0.64^c^	0.45^c^	0.46^c^
TII	0.69^c^	0.78^c^	0.65^c^	0.69^c^	0.70^c^	-	0.52^c^	0.47^c^	0.34^c^	0.50^c^	0.47^c^	0.36^c^	−0.15^a^	−0.39^c^	−0.39^c^	−0.25^b^	0.36^c^	0.32^c^	0.44^c^	0.46^c^
SFD	0.57^c^	0.63^c^	0.56^c^	0.70^c^	0.64^c^	0.77^c^	-	0.55^c^	0.38^c^	0.50^c^	0.42^c^	0.43^c^	−0.29^c^	−0.28^b^	−0.56^c^	−0.28^c^	0.43^c^	0.25^c^	0.55^c^	0.60^c^
IOB	0.52^c^	0.57^c^	0.45^c^	0.72^c^	0.57^c^	0.76^c^	0.82^c^	-	0.33^c^	0.57^c^	0.42^c^	0.37^c^	−0.08	−0.16	−0.30^c^	−0.16^a^	0.32^c^	0.21^c^	0.56^c^	0.47^c^
ETR	0.57^c^	0.61^c^	0.61^c^	0.56^c^	0.54^c^	0.60^c^	0.61^c^	0.63^c^	-	0.53^c^	0.63^c^	0.49^c^	−0.35^c^	−0.22^a^	−0.39^c^	−0.29^c^	0.31^c^	0.27^c^	0.36^c^	0.34^c^
DLC	0.68^c^	0.68^c^	0.58^c^	0.79^c^	0.58^c^	0.74^c^	0.75^c^	0.82^c^	0.75^c^	-	0.70^c^	0.43^c^	−0.26^c^	−0.28^b^	−0.41^c^	−0.26^c^	0.39^c^	0.28^c^	0.51^c^	0.52^c^
APC	0.64^c^	0.63^c^	0.58^c^	0.67^c^	0.55^c^	0.68^c^	0.63^c^	0.68^c^	0.78^c^	0.85^c^	-	0.39^c^	−0.24^c^	−0.27	−0.36^c^	−0.21^b^	0.46^c^	0.28^c^	0.52^c^	0.51^c^
IEF	0.49^c^	0.51^c^	0.67^c^	0.47^c^	0.68^c^	0.54^c^	0.62^c^	0.58^c^	0.64^c^	0.61^c^	0.54^c^	-	−0.48^c^	−0.24	−0.44^c^	−0.47^c^	0.32^c^	0.58^c^	0.45^c^	0.41^c^
IER	−0.34^c^	−0.37^c^	−0.56^c^	−0.27^c^	−0.54^c^	−0.31^c^	−0.40^c^	−0.29^c^	−0.45^c^	−0.40^c^	−0.36^c^	−0.61^c^	-	0.28^b^	0.28^c^	0.28^c^	0.28^c^	0.28^c^	0.28^c^	0.28^c^
WHE	−0.70^c^	−0.85^c^	−0.75^c^	−0.55^c^	−0.61^c^	−0.73^c^	−0.62^c^	−0.52^c^	−0.54^c^	−0.63^c^	−0.58^c^	−0.54^c^	0.50^c^	-	0.38^c^	0.34^b^	−0.31^c^	−0.18	−0.22	−0.19
SGN	−0.53^c^	−0.57^c^	−0.57^c^	−0.53^c^	−0.53^c^	−0.60^c^	−0.76^c^	−0.60^c^	−0.60^c^	−0.65^c^	−0.56^c^	−0.62^c^	0.55^c^	0.70^c^	-	0.55^c^	−0.30^c^	−0.23^b^	−0.35^c^	−0.40^c^
SBR	−0.44^c^	−0.49^c^	−0.63^c^	−0.41^c^	−0.54^c^	−0.54^c^	−0.57^c^	−0.47^c^	−0.54^c^	−0.54^c^	−0.48^c^	−0.68^c^	0.61^c^	0.65^c^	0.81^c^	-	−0.10	−0.25^b^	−0.14	−0.16^a^
TKL	0.52^c^	0.59^c^	0.54^c^	0.56^c^	0.57^c^	0.62^c^	0.63^c^	0.60^c^	0.53^c^	0.62^c^	0.61^c^	0.50^c^	−0.35^c^	−0.58^c^	−0.50^c^	−0.37^c^	-	0.36^c^	0.62^c^	0.56^c^
RAL	0.56^c^	0.60^c^	0.70^c^	0.51^c^	0.84^c^	0.63^c^	0.60^c^	0.56^c^	0.58^c^	0.60^c^	0.55^c^	0.79^c^	−0.66^c^	−0.59^c^	−0.55^c^	−0.59^c^	0.62^c^	-	0.44^c^	0.43^c^
CNL	0.52^c^	0.57^c^	0.54^c^	0.66^c^	0.65^c^	0.67^c^	0.75^c^	0.80^c^	0.60^c^	0.74^c^	0.69^c^	0.61^c^	−0.39^c^	−0.55^c^	−0.59^c^	−0.47^c^	0.79^c^	0.70^c^	-	0.65^c^
NWL	0.48^c^	0.53^c^	0.46^c^	0.65^c^	0.62^c^	0.67^c^	0.75^c^	0.74^c^	0.54^c^	0.70^c^	0.64^c^	0.55^c^	−0.36^c^	−0.50^c^	−0.58^c^	−0.44^c^	0.72^c^	0.64^c^	0.79^c^	-

QLW, quality of work; QNW, quantity of work; REA, rule adherence; TNP, technical performance; HPB, helpful behaviours; TII, taking initiative; SFD, self-development; IOB, innovative behaviours; ETR, emotional resilience; DLC, dealing with complexity; APC, adapting to crises; IEF, interpersonal flexibility; TKL, task-orientated leadership; RAL, relations-orientated leadership; CNL, change-orientated leadership; NWL, network-orientated leadership; IER, interpersonal rudeness; WHE, withholding effort; SGN, stagnation; SBR, stubborn resistance.

a , *p* ≤ 0.05;

b , *p* ≤ 0.01;

c , *p* ≤ 0.001.

The decision to retain the ESEM solution was supported when comparing the bifactor-ESEM solution to the bifactor-CFA and hierarchical-CFA solutions. An important question in selecting the optimal solution is whether the ESEM or the bifactor ESEM should be retained, given their almost identical fit. An examination of the parameter estimates (i.e. factor loadings) guided the decision-making process. Table S2 (Online Appendix 1) reveals a well-defined general factor, with positive loadings associated with positive work performance behaviours ESEM (|λ| = 0.41 to 0.80; *M* = 0.71) and negative loadings associated with counterproductive work behaviours (|λ| = –0.37 to –0.71; *M* = –0.58). All specific factors retained meaningful specificity (|λ| = 0.16 to 0.72; *M* = 0.39) after accounting for the variance explained by the general performance factor. The cross-loadings were generally very small (|λ| = –0.25 to 0.28; *M* = 0.10). Although the hierarchical-ESEM model had a slightly better fit, bifactor models are more effective in accounting for psychometric multidimensionality (Reise, as cited by Morin, 2023). This conclusion stems from the constraints inherent in hierarchical models and the criticism that these constraints are neither feasible in practice (Morin et al., 2016; Reise, 2012) nor substantively interpretable (Gignac, 2016). These constraints are not feasible because researchers cannot create items whose general factor-related variance is entirely mediated by the relevant primary factor (Gignac, 2008). The conclusion is that one can deduce (from the almost perfect fit of both the bifactor- and hierarchical-ESEM models) that an overarching global performance factor exists.

Several bifactor indices are reported in Table 4. Similar indices are recommended by Van Zyl and Ten Klooster (2022). The results indicated that the general factor explained 65% of the common variance extracted, with 35% spread across group factors. An ECV of 0.70 or more means that researchers should consider specifying a unidimensional model (Reise et al., 2013).^4^ The results also indicated that the omega coefficients exceeded 0.70. However, if one accounts for the reliable variance attributable to the general factor, the specific factors did not produce adequate omega coefficients (ω < 0.70). This means that the total performance scores were ‘essentially unidimensional’ (Rodriguez et al., 2016). However, Morin (2023) cautions against using ω_h_ and ω_hs_, as both tend to underestimate the reliability of the factors. Based on these observations, the bifactor-ESEM solution was retained, supporting H_1_.

**TABLE 4:** Reliability estimates and explained common variance.

Dimension	ECV	ω	ω_h(s)_
General factor	0.65	0.98	0.93
Quality of work	0.02	0.75	0.34
Quantity of work	0.01	0.76	0.23
Rule adherence	0.02	0.72	0.31
Technical performance	0.02	0.90	0.34
Helpful behaviours	0.02	0.85	0.35
Taking initiative	0.01	0.81	0.23
Self-development	0.01	0.81	0.25
Innovative behaviours	0.02	0.85	0.28
Emotional resilience	0.02	0.86	0.38
Dealing with complexity	0.01	0.74	0.16
Adapting to crises	0.02	0.88	0.30
Interpersonal flexibility	0.02	0.86	0.39
Task-orientated leadership	0.03	0.93	0.41
Relations-orientated leadership	0.01	0.79	0.26
Change-orientated leadership	0.01	0.85	0.25
Network-orientated leadership	0.02	0.81	0.24
Interpersonal rudeness	0.03	0.87	0.63
Withholding effort	0.01	0.74	0.28
Stagnation	0.02	0.78	0.29
Stubborn resistance	0.02	0.84	0.44

ECV, explained common variance; ω, model-based omega composite reliability; ω_h(s)_, hierarchical omega (specific factors).

In a new data set, the exported factor scores were combined with tenure and job level for the criterion-validity analysis. We correlated both tenure and job level with the general factor. Results indicated that tenure (*r* = 0.06; *p* = 0.33) was unrelated to performance, whereas job level (*r* = 0.28; *p* < 0.001) was positively related to performance, with a small (bordering medium) effect size. These results provide support for H_2B_ but not for H_2A_.

## Discussion

The first aim of this study was to determine the feasibility of drawing valid inferences regarding employees’ positions on a general performance factor based on their responses to the items within the IWPR. Evidence presented in this study suggests the presence of a general factor of performance in addition to narrow factors of performance, in line with the findings of a meta-analysis conducted by Viswesvaran et al. (2005). However, this does not mean that the narrow performance dimensions are meaningless in the presence of a general factor. The narrow dimensions still explained a meaningful amount of common variance in the same set of items and displayed a sufficient level of discriminant validity based on the inter-factor correlations. Carpini et al. (2017) argue that, in addition to a strong general factor, specific narrower dimensions could help to clarify what is meant with ‘performance’, where a general factor might appear as a vaguer term when trying to provide performance feedback. As phrased in the literature review, narrow dimensions provide a more nuanced or qualitatively rich understanding of the specific actions that employees could take to increase their performance. A general factor, simultaneously, serves as a justification to calculate an overall quantitative score, to differentiate employees and relate performance to larger unit level outcomes, such as the return on investment of selection processes or performance development interventions.

The weights given to dimensions in overall scores are often the result of implicit assumptions held by raters rather than being based on desired behaviours explicitly reinforced by the organisation’s decision-makers. Rotundo and Sackett (2002) found that the policies implemented by subject matter experts to determine the importance of different broad dimensions of performance for overall performance varied and that such variation was not affected by demographic variables. Instead, it appeared that factors such as what the raters observed, access to information on performance, and expertise on the topic of interest were more important. Rotundo and Sackett (2002, p. 66) were able to, based on hierarchical cluster analysis, group the evaluations of subject matter experts into three clusters, namely ‘(a) task performance weighted highest, (b) counterproductive performance weighted highest, and (c) equal and large weights given to task and counterproductive performance’. Rotundo and Sackett (2002) highlight that, depending on the weights given to, for example, task- or counterproductive performance, the predictive validity of psychological variables could differ markedly and that this matters in decision-making. The researchers of this study recommend that an explicit and considered weighting strategy be used as empirical research continues to emerge on the IWPR to reinforce a more uniform understanding of the construct in question across performance studies and a fair process in evaluating individual work performance.

The second aim of the present study was to determine whether biographical variables are corollaries of general work performance. Tenure did not appear to be a corollary of general individual work performance. The effect of tenure on performance seems to taper off after 5 years of job experience when the acquisition of knowledge and skills also decreases (Schmidt & Hunter, 1992; Schmidt et al., 2016). The mean tenure of participants in the present subset of data was 7.81 years, which might explain why a negligible correlation was found. A more recent meta-analytical study further revealed that tenure had a marginal effect on job performance (Sackett et al., 2022), which the present research supports.

In contrast to tenure, job level appears to be related to general individual work performance in the employee’s current position. Educational attainment and succession to more senior roles among the participants appeared to translate into greater performance. Caution should still be applied when interpreting this finding, as interactive variables, such as general cognitive ability, were not considered in this study. Job level might be a proximate variable of complexity, one that moderates the relationship between general cognitive ability and job performance (Salgado & Moscoso, 2019).

### Limitations and recommendations for future research

Sum scores, derived from summing or averaging responses on items, are rough approximations suitable for broad purposes. In such calculations, practitioners (or researchers) assume that all item loadings and their error variances are equal; therefore, the total score is a unit-weighted one. This contrasts with a ‘factor score’ derived from a congeneric model in which these assumptions are relaxed (McNeish & Wolf, 2020). Although sum scores are acceptable when the general factor derived from a bifactor model is reliable (Rodriguez et al., 2016) and when factor loadings (on both the specific and general factors) do not vary extensively, Table S2 shows that there are differences in the factor loadings. Consequently, the assumption of equal factor loadings is violated. For research purposes (where advanced applications are implemented and more precision is needed), we would thus recommend the differential weighting of items (i.e. weighted general scores) in line with McNeish and Wolf’s (2020) recommendations and the validation evidence presented in the current study.

In this study, performance reviews based on the IWPR were limited to direct managers to obtain credible ratings of performance (Myburgh, 2013; Schepers, 2008). Studies conducted to date suggest that rating sources could affect performance measures’ psychometric properties (Conway & Huffcutt, 1997; Heidemeier & Moser, 2009; Van Lill & Van Der Merwe, 2022). Therefore, the present study’s results can only serve as preliminary evidence in establishing the structure of a general factor. Future studies could inspect the general factor model’s inter-rater reliability and measurement invariance if the IWPR is completed by different raters, including the individual being rated, subordinates, and peers (Scullen et al., 2003).

Viswesvaran et al. (2005) argue that the presence of a general factor might be attributed to the presence of strong general factors in antecedents of performance, such as general mental ability or, as revealed in the meta-analysis of Sackett et al. (2022), personality-based integrity. This study only focussed on biographical variables as correlates of general individual work performance. Future studies could inspect the predictive validity of general mental ability or personality-based integrity to build out the nomological network surrounding general individual work performance. There is a paucity of literature regarding the outcomes of individual work performance (Carpini et al., 2017). While it was not the aim to inspect the outcomes of general work performance, future studies could inspect the predictive validity of performance for outcomes related to unit effectiveness, such as production and efficiency, market share and/or standing, and future growth (Seland & Theron, 2021). Finally, the biographical variables were assumed to have linear relationships with general job performance. However, tenure or job complexity might be curvilinearly related to job performance, which might be an interesting avenue for future research.

## Footnotes

1 It is important to distinguish the IWPQ from the IWPR in this study. Whereas the work on the IWPQ focussed on the cross-cultural applicability of a performance measure developed in the Netherlands, this study is an ongoing effort to investigate the factor structure of the locally (South African) developed IWPR performance measure.

2 A second-order model could possibly be converted into a bifactor approximation if one applies the Schmid and Leiman (1957) transformation procedure (SLP). However, given the limitations of the SLP procedure (see Reise et al., 2010), this study estimated an empirical bifactor model rather than a mathematically transformed model.

3 A factor loading of 0.30 indicates a small cross-loading (simple structure), and those ≥ 0.30 indicate meaningful cross-loadings (complex structure) (Morin et al., 2020).

4 Although the ECV was below 0.70, a unidimensional model was specified, which yielded a bad fit to the data (SB-χ^2^ = 18692.95; *df* = 3080; CFI = 0.54; TLI = 0.53; RMSEA = 0.11 [0.105, 0.108]; *p* = < 0.001; SRMR = 0.09).
